# Supplementary material for: MME and PTPRC: key renal biomarkers in lupus nephritis
Source: PeerJ. 2024 Sep 16;12:e18070. doi: 10.7717/peerj.18070 (PMC11412223; doi:10.7717/peerj.18070)
Supplement: Table S1 [file peerj-12-18070-s001.docx]

**Supplementary Table 1: Primers of the qRT-PCT.**

| Gene name | Primer | |
| --- | --- | --- |
| MX1 | forward primer | AGTAAGTGCAGCTGGTTCCT |
|  | reverse primer | AAGGATCATTGCACAGCGAC |
| MME | forward primer | CTTTCCGCAAGGCCCTTTAT |
|  | reverse primer | CCCACAGCATTCTCCATATTCC |
| EGR1 | forward primer | TCGAATCTGCATGCGTAACT |
|  | reverse primer | GCAAACTTCCTCCCACAAATG |
| PTPRC | forward primer | GCAGGGTCCACCTACATAAAT |
|  | reverse primer | TCATCCTCCAGAAGTCATCAAC |
| RORC | forward primer | CACTGAGGCCATTCAGTATGT |
|  | reverse primer | TGCTCCTGCTTTCAGTAGTATG |
| ACTB | forward primer | GAGGTATCCTGACCCTGAAGTA |
|  | reverse primer | CACACGCAGCTCATTGTAGA |
