# Supplementary material for: MME and PTPRC: key renal biomarkers in lupus nephritis
Source: PeerJ. 2024 Sep 16;12:e18070. doi: 10.7717/peerj.18070 (PMC11412223; doi:10.7717/peerj.18070)

**Sample sizes of 6 Lupus datasets (265 samples) (Up/Down)**

1. **Up expression datasets: Nephroseq Source:** [**http://www.nephroseq.org/resource/main.html#ac:1N10763,1N10758,1N10760,1N10958,1N10959,1N10761,1N10762,1N10960,1N10759,1N11029,1N10985,1N10983,1N10984,1N10998,1N10997,1N10986,1N10996,1N10995,1N10582,1N10586,1N10581,1N10576,1N10972,1N10578,1N10587,1N10580,1N10584,1N10579,1N10577,1N10583,1N10575,1N10585,1N11028,1N10979,1N10981,1N10980,1N10993,1N10982,1N10991,1N10992,1N10777,1N10778,1N10961,1N10962,1N10781,1N10779,1N10963,1N10780,1N10782,1N10994,1N10965,1N10968,1N10971,1N10496,1N10492,1N10964,1N10494,1N10488,1N10967,1N10969,1N10970,1N10966;cso:sizeSmallLarge;cv:detail;dso:geneOverex;ec:[1N2];epv:1N1.1N3,1N21,1N41;et:over;g:5788;pg:1;pvf:10992,12143,28059,28707,32650,33638,39659,1N800101741,1N800101748,1N800101756;scr:datasets;th:g100.0,p1.000,fc0.0;v:17**](http://www.nephroseq.org/resource/main.html#ac:1N10763,1N10758,1N10760,1N10958,1N10959,1N10761,1N10762,1N10960,1N10759,1N11029,1N10985,1N10983,1N10984,1N10998,1N10997,1N10986,1N10996,1N10995,1N10582,1N10586,1N10581,1N10576,1N10972,1N10578,1N10587,1N10580,1N10584,1N10579,1N10577,1N10583,1N10575,1N10585,1N11028,1N10979,1N10981,1N10980,1N10993,1N10982,1N10991,1N10992,1N10777,1N10778,1N10961,1N10962,1N10781,1N10779,1N10963,1N10780,1N10782,1N10994,1N10965,1N10968,1N10971,1N10496,1N10492,1N10964,1N10494,1N10488,1N10967,1N10969,1N10970,1N10966;cso:sizeSmallLarge;cv:detail;dso:geneOverex;ec:[1N2];epv:1N1.1N3,1N21,1N41;et:over;g:5788;pg:1;pvf:10992,12143,28059,28707,32650,33638,39659,1N800101741,1N800101748,1N800101756;scr:datasets;th:g100.0,p1.000,fc0.0;v:17)
2. **Down expression datasets: Nephroseq Source:** [**http://www.nephroseq.org/resource/main.html#ac:1N10763,1N10758,1N10760,1N10958,1N10959,1N10761,1N10762,1N10960,1N10759,1N11029,1N10985,1N10983,1N10984,1N10998,1N10997,1N10986,1N10996,1N10995,1N10582,1N10586,1N10581,1N10576,1N10972,1N10578,1N10587,1N10580,1N10584,1N10579,1N10577,1N10583,1N10575,1N10585,1N11028,1N10979,1N10981,1N10980,1N10993,1N10982,1N10991,1N10992,1N10777,1N10778,1N10961,1N10962,1N10781,1N10779,1N10963,1N10780,1N10782,1N10994,1N10965,1N10968,1N10971,1N10496,1N10492,1N10964,1N10494,1N10488,1N10967,1N10969,1N10970,1N10966;cso:sizeSmallLarge;cv:detail;dso:geneOverex;ec:[1N2];epv:1N1.1N3,1N21,1N41;et:under;g:4599;pg:1;pvf:10992,12143,28059,28707,32650,33638,39659,1N800101741,1N800101748,1N800101756;scr:datasets;th:g100.0,p1.000,fc0.0;v:17**](http://www.nephroseq.org/resource/main.html#ac:1N10763,1N10758,1N10760,1N10958,1N10959,1N10761,1N10762,1N10960,1N10759,1N11029,1N10985,1N10983,1N10984,1N10998,1N10997,1N10986,1N10996,1N10995,1N10582,1N10586,1N10581,1N10576,1N10972,1N10578,1N10587,1N10580,1N10584,1N10579,1N10577,1N10583,1N10575,1N10585,1N11028,1N10979,1N10981,1N10980,1N10993,1N10982,1N10991,1N10992,1N10777,1N10778,1N10961,1N10962,1N10781,1N10779,1N10963,1N10780,1N10782,1N10994,1N10965,1N10968,1N10971,1N10496,1N10492,1N10964,1N10494,1N10488,1N10967,1N10969,1N10970,1N10966;cso:sizeSmallLarge;cv:detail;dso:geneOverex;ec:[1N2];epv:1N1.1N3,1N21,1N41;et:under;g:4599;pg:1;pvf:10992,12143,28059,28707,32650,33638,39659,1N800101741,1N800101748,1N800101756;scr:datasets;th:g100.0,p1.000,fc0.0;v:17)

**6 Lupus datasets references**

1. **Berthier Lupus Glom** (n=46)^[1]^
2. **Berthier Lupus Mouse Kidney**(n=68)^[1]^
3. **Berthier Lupus TubIntn** (n=47)^[1]^
4. **ERCB Lupus TubInt**(n=41)^[2]^
5. **ERCB Lupus Glom**(n=32)^[3]^
6. **Peterson Lupus Glom**(n=31)^[4]^

[1] Berthier CC, Bethunaickan R, Gonzalez-Rivera T, et al. Cross-species transcriptional network analysis defines shared inflammatory responses in murine and human lupus nephritis(J). J Immunol. 2012.189(2):988-1001

[2] ERCB. ERCB Lupus TubInt Dataset Summary [https://nephroseq.org/resource/ui/component/dataset.html?component=d:1N156636801[J](https://nephroseq.org/resource/ui/component/dataset.html?component=d:1N156636801%5bJ)]. Journal 2018(Issue).

[3] ERCB. ERCB Lupus Glom Dataset Summary <https://nephroseq.org/resource/ui/component/dataset.html?component=d:1N156636800(J>). 2018

[4] Peterson KS, Huang J-F, Zhu J, et al. Characterization of heterogeneity in the molecular pathogenesis of lupus nephritis from transcriptional profiles of laser-captured glomeruli(J). Journal of Clinical Investigation. 2004.113(12):1722-33


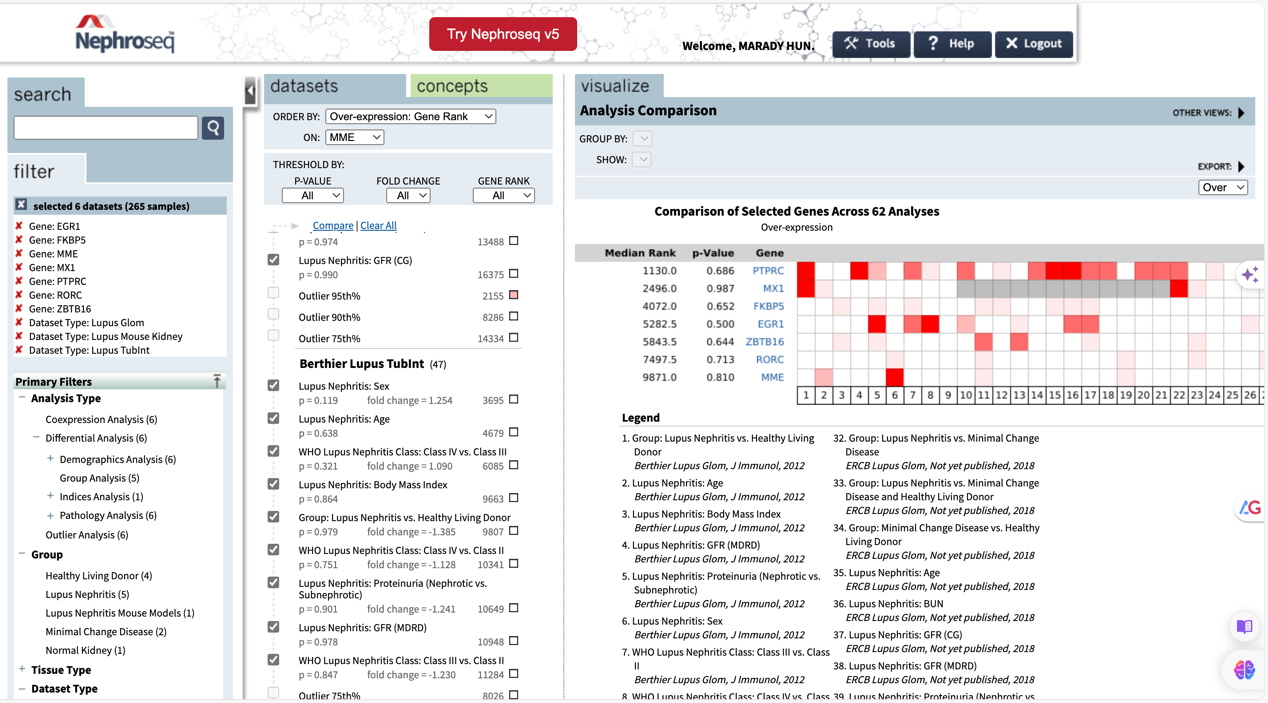

Supplement: Table S2 — Nephroseq datasets and Lupus datasets. [file peerj-12-18070-s002.docx]
